# Triacylglycerols sequester monotopic membrane proteins to lipid droplets

**DOI:** 10.1038/s41467-020-17585-8

**Published:** 2020-08-07

**Authors:** Lucie Caillon, Vincent Nieto, Pauline Gehan, Mohyeddine Omrane, Nicolas Rodriguez, Luca Monticelli, Abdou Rachid Thiam

**Affiliations:** 1 Laboratoire de Physique de l’École Normale Supérieure, ENS, Université PSL, CNRS, Sorbonne Université, Université de Paris, F-75005 Paris, France; 2 University of Lyon, CNRS, Molecular Microbiology and Structural Biochemistry (MMSB, UMR 5086), F-69007 Lyon, France; 3 Sorbonne Universités, UPMC Univ Paris 06, Ecole Normale Supérieure, CNRS, Laboratoire des Biomolécules (LBM), 4 place Jussieu, 75005 Paris, France; 4 Département de Chimie, Ecole Normale Supérieure, PSL Research University, UPMC Univ Paris 06, CNRS, Laboratoire des Biomolécules (LBM), Paris, France

**Keywords:** Membrane biophysics, Biological physics

## Abstract

Triacylglycerols (TG) are synthesized at the endoplasmic reticulum (ER) bilayer and packaged into organelles called lipid droplets (LDs). LDs are covered by a single phospholipid monolayer contiguous with the ER bilayer. This connection is used by several monotopic integral membrane proteins, with hydrophobic membrane association domains (HDs), to diffuse between the organelles. However, how proteins partition between ER and LDs is not understood. Here, we employed synthetic model systems and found that HD-containing proteins strongly prefer monolayers and returning to the bilayer is unfavorable. This preference for monolayers is due to a higher affinity of HDs for TG over membrane phospholipids. Protein distribution is regulated by PC/PE ratio via alterations in monolayer packing and HD-TG interaction. Thus, HD-containing proteins appear to non-specifically accumulate to the LD surface. In cells, protein editing mechanisms at the ER membrane would be necessary to prevent unspecific relocation of HD-containing proteins to LDs.

## Abstract (web summary)

Triacylglycerols (TG) are synthesized at the endoplasmic reticulum (ER) bilayer and packaged into monolayer lipid droplets (LDs), but how proteins partition between ER and LDs is poorly understood. Here authors use synthetic model systems and find that proteins containing hydrophobic membrane association domains strongly prefer monolayers and that returning to the bilayer is unfavorable.

## Introduction

**Fig. 1:**
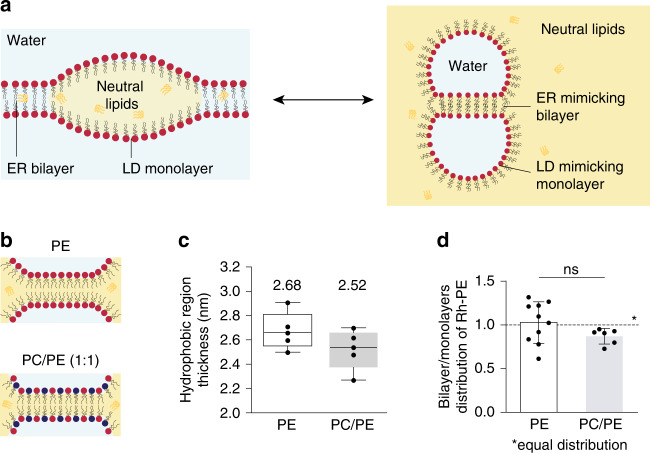
Characterization of droplet interface bilayers. **a** Schematic representation of the ER phospholipid bilayer contiguous with the monolayer of a nascent LD (left side); the corresponding DIB system reproducing contiguous bilayer and monolayers is shown on the right side. The water phase is represented in light blue and the oil phase in yellow (neutral lipid, e.g. triglycerides (TG)). **b** Drawings of a DIB bilayer of DOPE (top) and DOPC/DOPE (1:1) (bottom). **c** The thickness of the hydrophobic region of the DIB bilayer in DOPE (white) and DOPC/DOPE (1:1) (gray) is determined by capacitance measurement. Results are shown as box-plots (box limits, upper and lower quartiles; middle line, median; whiskers, minimum and maximum value; the mean is indicated) from *n* = 5 independent experiments. Each point is represented as a black dot. **d** Distribution of Rh-PE between the bilayer and the monolayers in DOPE (white) and DOPC/DOPE (1:1) (gray) DIBs. The results are the mean ± SD of respectively *n* = 10 and *n* = 5 independent measurements. Each point is represented as a black dot. Significance was determined by Welch’s *t*-test (unpaired parametric test, two-tailed *p*-value) and is indicated by ns (not significant): *p* > 0.05. Source data are provided as a Source Data file.

Lipid droplets (LDs) are lipid storage organelles primarily functioning in cellular energy metabolism^1^. LD biogenesis occurs at the endoplasmic reticulum (ER) membrane during energy rich or stress conditions. LD biogenesis starts with the synthesis of neutral lipids, such as triacylglycerols (TG) or sterol esters, which, at low concentration, are dissolved in the ER bilayer^2^. Upon increase in concentration, neutral lipids demix from membrane phospholipids to form an oil lens or a nascent droplet within the bilayer^3^ (Fig. 1a). The lens grows and emerges in the cytosol as a mature LD: an oil-in-water droplet covered by a phospholipid monolayer with proteins embedded. Indeed, throughout the steps of LD emergence, many proteins target to the surface and around the LD^4–7^. Proteins targeting the LD surface essentially come from the ER membrane or from the cytosol^7^, and ensure proper LD budding^8^. How proteins bind and accumulate to LDs is not well understood but the neutral lipid chemistry is determinant to these processes^9^. Specificity of protein targeting to LDs is at the heart of LD biology, and understanding its principles will provide fundamental knowledge on lipid metabolism and cellular proteostasis^5–7,10,11^.

Membrane physicochemical properties regulate the protein distribution at bilayer-encircled organelles^12–14^. The LD-water interface is distinguishable from a bilayer-water interface by several features: it can sustain a loose lipid packing^9,15,16^; the thickness of the underlying hydrophobic region, up to hundreds nm, is much larger than the hydrophobic thickness of a bilayer (~3 nm)^17,18^; the hydrophobic core consists of neutral lipids, instead of phospholipid acyl chains. Considering these discrepancies in physical chemistry, it may not be surprising that proteins show preference for one interface over the other.

Most proteins physically associating with LD surfaces are either peripheral or monotopic^6,7,11^ and do not fully cross bilayer membranes. Proteins moving from the ER to LD surface, contain helical hydrophobic domains (HDs), which are monotopic integral membrane domains embedded only in one face of the membrane. These HDs include helical hairpins, hydrophobic helices, and possibly transmembrane domains not fully crossing a bilayer^11,19–21^. In contrast, soluble proteins often use amphipathic helices (AHs) for binding to LDs.

The binding of AHs to LDs is more documented both in vitro and in vivo^9,16,19,22–24^: AHs act as surfactants, favorably adsorbed to the oil/water interface of LDs to decrease the interfacial energy. AHs recognizes a variety of membrane features, such as surface charges, curvature, phospholipid packing defects, and neutral lipids^9,16,19,22,23^. In contrast, much less is known about HDs which target to LDs mostly from the ER membrane through ER-LD connecting bridges^25–27^. Neither the energetics involved in their binding to LDs nor the parameters controlling their ER-to-LD partitioning are known.

The inclusion of HD-containing proteins into lipid bilayers can cause local perturbation to the bilayer properties, which translates into an energy penalty^28–31^. For instance, proteins can locally perturb the organization of the phospholipids and enhance exposure of the hydrophobic core of the bilayer to water^28–32^. The extent of membrane perturbation depends on the amino acid sequence, and is for instance important when the mismatch between the bilayer thickness and the HD length is significant^31,32^. As for protein insertion into LD surfaces, no information is available regarding the energy cost of the process, nor the type and the extent of the perturbation generated in the surrounding lipids.

Here, we study how LD proteins, and particularly monotopic HD-containing proteins, partition between a bilayer and an LD in contiguity. We employ the droplet interface bilayer (DIB) system^33^ (Fig. 1a) to study the partitioning of proteins and peptides bearing HDs, as compared with AH-containing proteins. We find that all proteins investigated partition preferentially to the LD monolayer surface, but HD-containing proteins display a higher enrichment in the monolayer than AH-containing ones. Relocation of HD proteins to the bilayer is unfavorable, while moving from the bilayer to the monolayer is spontaneous. We also found that protein distribution is altered by the ratio between PC and PE phospholipids by regulating the extent of HD-TG contact at the LD surface.

## Results

### Characterization of the droplet interface bilayer system

To determine the partition coefficient of proteins capable of binding a monolayer and a bilayer in contiguity, we decided to employ the droplet interface bilayer (DIB) system^33,34^. DIBs consist of two micrometric buffer-in-oil droplets covered by a phospholipid monolayer (Fig. 1a). The oil phase used here was trioctanoate, a triglyceride with similar interfacial energy as triolein^8^, the major cellular neutral lipid. Contact of the droplets induces the formation of a bilayer in contiguity with the two monolayers (Fig. 1a). Thus DIBs mimic ER-LD contiguity (Fig. 1a) without curvature considerations; the different interfaces are flat at the protein scale and the concavity of the monolayer surfaces is irrelevant with respect to curvature. For phospholipids, we used dioleoyl phosphatidylethanolamine (termed PE) and dioleoyl phosphatidylcholine (termed PC) (Fig. 1b). Phospholipids were added to the oil phase and were recruited to the surface of the aqueous droplets whose contact generates within 5 min an equilibrated DIB^34,35^.

DIBs can be generated with almost any phospholipids^35^. In the case of non-bilayer phospholipids, such as DOPE, a PE-DIB bilayer is made thanks to the presence in the bilayer of TG molecules whose level is decreased by the addition of PC^35^. To get insight into the amount of TG present in a PE-DIB bilayer, we measured the thickness of the hydrophobic region of the bilayer by capacitance measurements^36^ (Fig. 1c, Supplementary Fig. S1a). The thickness measured in PE-DIBs was 2.68 nm, only ~7% above the thickness of a PC/PE (1:1) DIB, 2.52 nm (Fig. 1c). Importantly, these values are comparable to the thickness of the hydrophobic region in phospholipid vesicles devoid of oil, between 2.3–2.7 nm^18^. Additionally, all-atom molecular dynamics simulations indicate that adding PE to a PC bilayer devoid of oil is sufficient to increase bilayer thickness up to 10% (Supplementary Fig. S1b). Altogether, these data indicate that the thickness of the DIBs made here is similar to that of phospholipid bilayer vesicles and is not significantly affected by the presence of oil.

Since the PC/PE mixture was added to the oil phase, we wanted to know whether this bulk ratio reflects the monolayer composition. We had previously measured the surface tension of monolayers made of PC/PE and found a linear decrease as this ratio increased in bulk oil^35^ (from ~2 mN m^−1^ for PE at 100% to ~0.6 mN m^−1^ for 100% PC). This supports that the bulk PC/PE composition reflects the one at the monolayer, as otherwise a plateau of surface tension against PC/PE should be observed. We next asked whether the PC/PE ratio in the monolayer and in the DIB bilayer are identical. To address this, we measured the partitioning of Rhodamine-PE (Rh-PE) between the DIB monolayers and bilayer, in the case of PE and PC/PE (1:1) DIBs. In pure PE-DIB, Rh-PE was uniformly distributed, indicating that the distributions of Rh-PE and PE are similar. In PC/PE, Rh-PE was also almost uniformly distributed, suggesting that the monolayer and the bilayer have a similar PC/PE composition (Fig. 1d, Supplementary Fig. S1c). To confirm this finding, we investigated lipid distribution in model nascent LDs using molecular dynamics simulations, in three systems containing TG and (a) pure DOPC or DOPC/DOPE mixtures, (b) 80/20 and (c) 60/40. We found that DOPE and DOPC mix ideally and their distribution was approximately homogeneous (Supplementary Fig. S1d). DOPC was only slightly enriched in the monolayer compared to the bilayer, while DOPE was slightly enriched in the bilayer compared to the monolayer—the differences being minor in both cases (Supplementary Fig. S1d). Overall, the data confirm that PE/PC mixtures are ideal mixtures, with an approximately even distribution of both lipids between the monolayer and bilayer interfaces.

The above characterizations indicate that DIBs recapitulate sufficiently well conditions of a bilayer containing an oil droplet, as previously shown^35^. We subsequently use DIBs to study protein partitioning.

### Monotopic proteins strongly bind to TG-covering monolayers

**Fig. 2:**
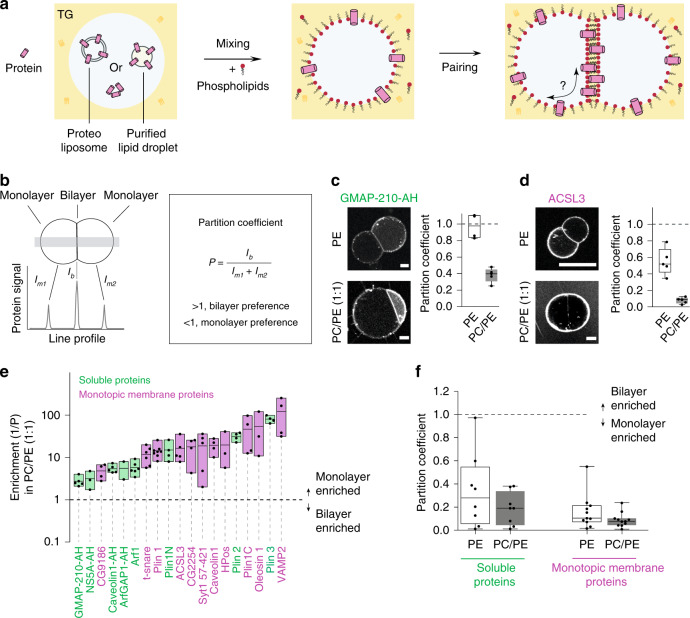
Partitioning of hydrophobic and amphipathic helix-containing proteins to droplet interface bilayers. **a** Formation of protein-containing DIBs: soluble protein, proteoliposome, or purified LDs are added to the buffer droplet (left). Mixing the droplet in a TG-containing phospholipids solution allows the relocalization of proteins to the buffer droplet interface (middle). When two drops come close together, their monolayers zip to form a bilayer. The proteins are thus offered to relocalize to the bilayer (right). **b** Protein distribution between the bilayer and the monolayer is determined by the partition coefficient P, which is the protein signal at the bilayer divided by sum of its signals at the monolayers. When a protein partitions preferentially to the bilayer, *p* > 1; if preference is for the monolayer then *p* < 1. **c**, **d** Distribution of GMAP-210-AH (soluble protein) and ACSL3 (monotopic membrane protein) respectively, in DOPE or DOPC/DOPE (1:1) DIBs. Scale bar: 20 µm. The partition coefficient is represented for each condition as box-plots from *n* = 5 independent measurements (excepted for GMAP-210-AH in PE, *n* = 4). **e** Enrichment parameter in DOPC/DOPE (1:1) membranes for AH- (green) and HD- (pink) containing proteins, shown as floating bars (bar limits, min to max values; central line, mean), 2 ≤ *n* ≤ 8 independent measurements were done for each protein. HD-containing proteins coming from LDs are Plin 1, Plin 1C, ACSL3, CG2254, CG9186, Oleosin 1, Caveolin 1, HPos; those coming from proteoliposomes are Syt1 57-421, t-snare, Vamp2; AH-containing proteins coming from LDs are Plin3, Plin2, Plin1N; the other AHs are added soluble. **f** Average partition coefficient of the groups of soluble or monotopic membrane proteins in DOPE or DOPC/DOPE (1:1). Results presented in (Supplementary Fig. S2e) were used to generate (**f**). Box-plots are defined as follow: box limits, upper and lower quartiles; middle line, median; whiskers, minimum and maximum value. In **c**, **d**, **e**, **f** each point is represented as a black dot. Source data are provided as a Source Data file.

We screened the monolayer-bilayer partitioning of two classes of proteins or peptides: soluble proteins, targeting to LDs from the cytosol, and monotopic integral membrane proteins (moving from the ER bilayer to LD surface. Soluble peptides were directly added to the buffer droplets. Monotopic membrane proteins were added to the buffer droplets from purified LDs or from proteoliposomes (Fig. 2a); mixing relocalized the proteins from LDs, or proteoliposomes, to the interface between the buffer droplet and the oil phase. Phospholipids from liposomes or LDs also relocalized to this new interface, but their total amount was always much less than the amount of the exogenous phospholipids we added; the latter would control the interfacial lipid composition in all of our systems. In practice, buffer-in-oil droplets containing the proteins at the interface were prepared before adding phospholipids to the oil phase (Fig. 2a). Two droplets were then brought together to form a DIB. The protein partition coefficient was determined 10 min after contact, at equilibrium, by quantifying the enrichment level of the protein in the bilayer relative to the monolayers (Fig. 2b).

For monotopic membrane proteins, we tested Plin1, ACSL3, CG2254, CG9186, oleosin 1, Hpos, and caveolin1 (Fig. 2d, Supplementary Fig. S2a, c), most of which contain helical hairpin and hydrophobic or amphipathic helix motifs responsible for their localization to LDs^37–40^. These proteins were tagged with fluorescent proteins and expressed in cells that were subsequently loaded with oleate to induce LDs. LDs bound by the proteins were purified and added to the DIB system. One limitation of this approach is that other proteins contained in the LDs would also relocalize to the DIB interfaces, although not visible. Furthermore, proteins with single transmembrane domains, not fully crossing the ER bilayer, could target to the LD surface, but this has never been shown clearly so far. To test this hypothesis, we prepared proteoliposomes containing some of the SNARE components bearing a transmembrane helix, but not crossing the bilayer. Finally, we studied a group of soluble proteins, including Plin2–3, Plin1 AH-containing domains^19,24^, and the lipid packing sensors ArfGap1-AH^41^ and GMAP-210-AH^42^ (Fig. 2c, Supplementary Fig. S2c, d).

For all of the tested proteins, we found a stronger partitioning to the monolayers than to the bilayer, independently of PC/PE ratio (Fig. 2e, f, Supplementary Fig. S2e, f). Additionally, HD-containing proteins showed on average a higher LD enrichment than AH proteins (Fig. 2e, Supplementary Fig. S2f), supporting that proteins coming from the ER bilayer better associate with LDs than soluble proteins. For a subgroup of HD proteins, we measured the partitioning in both PC/PE (1:1) and a more biologically relevant composition (DOPE/DOPC/liverPI/cholesterol, 5:3:1:1), and found very similar results (Supplementary Fig. S2a, b).

Finally, the bilayer localization of AH-containing proteins was increased by addition of PE in most cases, but partitioning to the monolayer region was still more favorable (Fig. 2f, Supplementary Fig. S2e, f). The negative spontaneous curvature of PE is known to cause lipid packing defects, which can be sensed by AHs^43^. This is well illustrated by the highest partition coefficient (close to 1) obtained in PE with the AH domains of GMAP-210 and ArfGap1, which are lipid packing sensors^43^ (Fig. 2c, Supplementary Fig. S2e). In contrast to AHs, the dependence of HD-containing protein on PC/PE was less clear (Supplementary Fig. S2e, f).

In summary, both AH- and HD-containing proteins localized preferentially to the monolayer interface over the bilayer. HD-proteins more strongly partitioned to the monolayer and barely relocated to the bilayer.

### KWALP peptides recap the global behavior of HD proteins

**Fig. 3:**
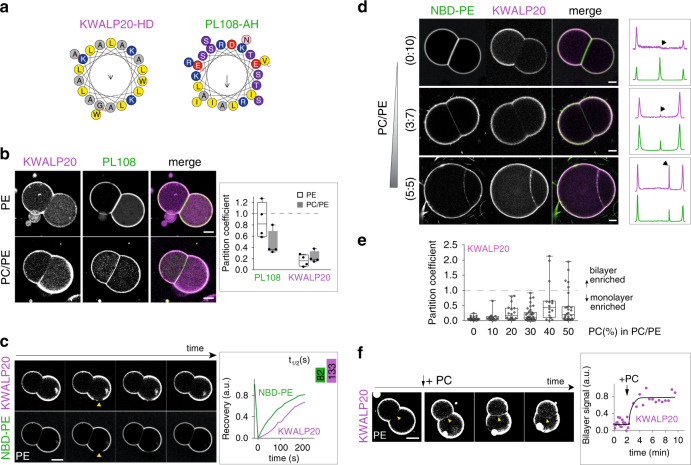
PC/PE ratio modulates the partitioning of model AH and HD peptides. **a** Helical wheel representation of KWALP20 and PL108-AH, generated from HeliQuest^69^. **b** Distribution of KWALP20 and PL108 in DOPE or DOPC/DOPE (1:1) DIBs. KWALP20 is labeled with and PL108 with NBD. Scale bar: 50 µm. The partition coefficient is plotted for both peptides as box-plots (box limits, upper and lower quartiles; middle line, median; whiskers, minimum and maximum value), from *n* = 4 independent measurements for each condition. Individual data are shown as black dots. **c** FRAP experiment shows that KWALP20 (purple) and lipid (green) signals are mobile at the monolayer. NBD-PE reports for phospholipids. Yellow arrows indicate the area bleached. Scale bar: 50 µm. Recovery half-time was obtained using one-phase association fitting in GraphPad software and is shown in the upper right box. **d** KWALP20 distribution in DIBs of different PC/PE ratio. Line profiles (not displayed) are drawn perpendicular to the bilayer and monolayers (as described in Fig. 2b); the thickness of the line is 30-40% of the bilayer size. The corresponding signals are shown in the box (right); black arrows depict the bilayer signal. Scale bar: 20 µm. **e** Partition coefficient of KWALP20 in DIBs of different DOPC/DOPE ratios represented as box-plots (box limits, upper and lower quartiles; middle line, median; whiskers, minimum and maximum value). Sample size was *n* = 31 for 0% PC, *n* = 14 for 10% PC, *n* = 18 for 20% PC, *n* = 41 for 30% PC, *n* = 16 for 40% PC and *n* = 28 for 50% PC. Each data point is plotted. **f** Relocalization of KWALP20 from the monolayer to the bilayer after addition of DOPC to DOPE DIBs. The bilayer signal is plotted over time. Image brightness is enhanced to improve bilayer viewing. Scale bar: 50 µm. Source data are provided as a Source Data file.

At this stage, it is difficult to explain the partitioning trend of the full-length HD proteins. This is in part because most of the proteins, coming from purified LDs, may interact with other unidentified proteins in the system. Also, LD proteins can bear multiple HDs and/or AHs; this is the case for Oleosin1, Caveolin1, HPos, and ACSL3^37,38,40^, which possess an AH motif adjacent to their HD motif. To better understand the determinants of partitioning for pure HD domains, we focused on model peptides of the KWALP family. KWALP peptides consist of a repeated leucine-alanine motif (Fig. 3a), bounded by two tryptophan residues at the C-terminus and three lysine residues at the N-terminus; to this N-terminus we added a glycine linked to a rhodamine-B dye. This peptide features a strong tendency towards helical conformation and transmembrane partitioning, as reviewed from numerous previous studies^28^; therefore they represent a valid model for transmembrane domains of proteins, including those localizing to the Golgi and plasma membranes^17,28,44^. Moreover, KWALP represents an excellent model for the minimal basic hydrophobic sequences, commonly found in HD domains of LD proteins (Supplementary Fig. S3). We used KWALP20, with 16 hydrophobic amino acids (Fig. 3a) and a length (when folded in an α-helix) close to the ER bilayer thickness (requiring ~ 20 hydrophobic amino acids^28^). For comparison, we also studied the partitioning of an AH motif derived from the 11-mer repeat of Perilipin1—termed here PL108 (Fig. 3a)^24^. KWALP20 and PL108 represent useful models for the two classes of HD and AH proteins tested above.

We prepared DIBs containing both KWALP20 and PL108 to compare their distribution under identical experimental conditions. When the DIB contained PE exclusively, PL108 partitioned almost equally between the monolayer and the bilayer (Fig. 3b), while KWALP20 was surprisingly absent from the bilayer (Fig. 3b). When PE/PC (1:1) was used, KWALP20 signal in the bilayer increased, while PL108 bilayer concentration significantly decreased (Fig. 3b). These observations are consistent with the behavior of most HD- and AH-containing proteins (Fig. 2e, Supplementary Fig. S2f): both peptides partition more favorably to the monolayer, especially for KWALP20; in PE lipids, PL108 partitions more evenly, like GM210-AH, and KWALP20 is barely detectable to the bilayer, like Oleosin1, Caveolin1 or Hpos (Supplementary Fig. S2c).

Since KWALP recapitulated the partitioning of most of the full-length HD-proteins (Fig. 2e, f, Supplementary Fig. S2c, e, f), we further investigated the driving forces for its distribution to establish general principles underpinning the enrichment of HDs to LD surface.

### PC/PE ratio regulates the partitioning of KWALP

KWALP20 was almost totally absent from the bilayer in PE, while it was well folded in the monolayer (Supplementary Fig. S4a). Importantly, the peptide was laterally mobile, as shown by the rapid recovery of fluorescence following photobleaching (Fig. 3c, Supplementary Fig. S4c); this recovery was indeed due to in-plane diffusion, because recovery from bulk did not happen within 10 minutes (Supplementary Fig. S4b). Since the hydrophobic thickness of the PE DIB bilayer is comparable to the peptide length, it is unlikely that KWALP20 localization to the bilayer was prevented by hydrophobic mismatch. We next increased further the PC/PE ratio, and observed that the concentration of the peptide in the bilayer increased with PC level, but it still remained lower than in the monolayer (Fig. 3d, e). In the bilayer, the KWALP peptide was also mobile but showed a significant tendency to cluster as the PC level was increased (Fig. 3f, Supplementary Fig. S4f). To verify if the bilayer localization was dynamic, and if diffusion was not prevented by peptide aggregation, we followed the peptide signal while changing the phospholipid composition. Starting from a pure PE DIB, where KWALP20 was absent from the bilayer, we added a TG solution containing PC to the oil phase surrounding the droplets (Fig. 3f). The recruitment of PC to the interface of the droplets was demonstrated by the increase in the contact angle between the droplets^35^ (Supplementary Fig. S4e) and it was concomitant with an increase of KWALP20 signal in the bilayer (Fig. 3f). We also noticed the appearance of KWALP clusters after PC recruitment (Fig. 3f, Supplementary Fig. S4f), suggesting that clustering is an inducible equilibrium state. For comparison, PL108 followed the opposite trend, as it was excluded from both the bilayer and the monolayer by PC recruitment (Supplementary Fig. S4g). These results suggest that the system has lower free energy when the peptide is at the monolayer, and the free energy gap between configurations where KWALP is at the monolayer or at the bilayer is decreased by PC. This energy gap does not come from a hydrophobic mismatch since when we repeated the above experiments with a longer KWALP version, namely KWALP28 (Supplementary Fig. S3a), which should be longer than the bilayer thickness, the peptide behave almost exactly as KWALP20, within the resolution limits of our measurements (Supplementary Fig. S4c, d).

In conclusion, our results show that KWALP20 partitions dynamically between the monolayer and the bilayer, but it has a clear preference for the monolayer, especially in high PE levels. In cells, if there is no regulation of ER-to-LD partitioning, HDs would be favorably adsorbed at the LD surface as a result of free energy minimization.

### Phospholipid shape defines KWALP partitioning

Since changing PC/PE ratio varied partitioning, we hypothesized that the affinity of HDs for lipids may be a driving force for partitioning. An HD protein can interact with phospholipid acyl chains, TG, and water, although interactions with the latter are unfavorable. We wanted to know which interactions would be responsible for the accumulation of the peptides to the monolayer.

**Fig. 4:**
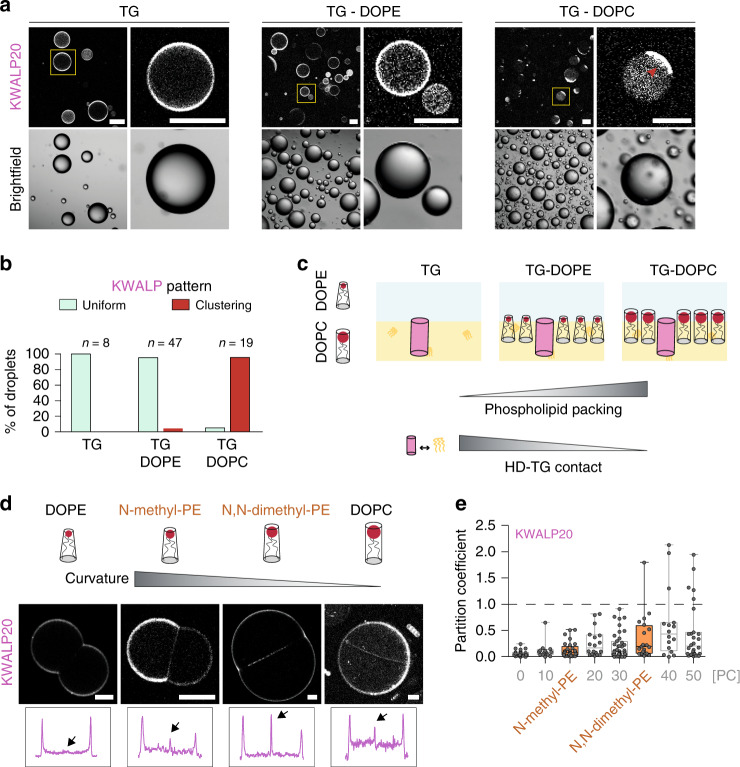
The partitioning of KWALP is altered by phospholipid shape. **a** KWALP20 surface distribution in bare TG-buffer droplets or in TG-buffer droplets covered by DOPE or DOPC. Red arrow highlights peptide clustering (in DOPC condition). The yellow square regions are enlarged on the right side of each image. Scale bar: 100 µm. **b** Quantification of KWALP pattern, i.e., uniform (light green) or clustering (red) signal, in TG (*n* = 8), TG + DOPE (*n* = 47) and TG + DOPC (*n* = 19), from *n* independent measurements. **c** Schematic representation of the difference in phospholipid packing, and thus in HD-TG contact, when DOPE (cone shape) or DOPC (cylinder shape) are present. Increasing DOPC concentration in a DOPC/DOPE monolayer increases the lipid packing and decreases the contact between HDs and TG. **d** Distribution of KWALP20 in DOPE, *N*-methyl-PE, *N*,*N*-dimethyl-PE, and DOPC DIBs. These phospholipids have incremental curvature between that of DOPE and DOPC. KWALP20 is labeled with Rh-B. Line profiles (not displayed) are drawn perpendicular to the bilayer and monolayers (as described in Fig. 2b); the thickness of the line is 30–40% of the bilayer size. Arrows indicate the bilayer signal. Scale bar: 20 µm. **e** Partition coefficient of KWALP20 in DIBs of different compositions is represented as box-plots (box limits, upper and lower quartiles; middle line, median; whiskers, minimum and maximum value). Sample size is *n* = 29 and 20 for KWALP20 in *N*-methyl-PE and *N*,*N*-dimethyl-PE respectively. Previous results of varying PC/PE ratios (Fig. 3e) are reported in light gray. Individual data points are indicated. Source data are provided as a Source Data file.

We prepared phospholipid-free buffer-in-TG droplets containing KWALP20. The protein signal at the interface was uniform (Fig. 4a, b). When the interface was lined by PE, the signal was also uniform in most cases (Fig. 4a, b). Instead, when PC alone lined the interface, the protein formed clusters (Fig. 4a, b) in which the peptide was mobile (Supplementary Fig. S5a). Similar clustering was observed when PC was added to DIBs (Fig. 3f, Supplementary Fig. S4f), and never observed for AHs. Apparently, protein-protein interactions become more favorable at the PC monolayer interface, suggesting that KWALP has more affinity for TG than for phospholipid acyl chains. We thus hypothesize that the relative contact of an HD with TG and phospholipids determines HD monolayer-bilayer partitioning.

The monolayer of a droplet contiguous with a bilayer is less packed with phospholipids than the bilayer leaflets^9^. In a bilayer, HD peptides would be in contact with phospholipid acyl chains along their full length while, in a monolayer, a significant fraction of the peptide would be in contact with TG. Thus, the higher affinity of HD proteins for TG over phospholipids can explain why HD proteins partition more favorably to monolayers. Why would the PC/PE ratio matter in this picture? Very likely because PC/PE can modulate the probability for HDs to contact with TG. PC and PE do not differ in their acyl chain composition (two oleoyl chains in both cases) but they differ in their average shape: PC has a cylindrical shape while PE is conical (Fig. 4c). Therefore, PC proffers a higher phospholipid monolayer packing which, in turn, reduces the probability of contact of an HD with TGs, and increases the probability of contact with phospholipid acyl chains (Fig. 4c). As a consequence, HD would less efficiently partition to the monolayer when the PC/PE ratio is increased.

Our model suggests that phospholipid shape modulates HD monolayer-bilayer partitioning. To test this, we used dioleoyl phosphatidic acid (PA), which has a negative spontaneous curvature, like PE. We found that, in a PA DIB, KWALP20 was almost excluded from the bilayer (Supplementary Fig. S5b), as observed in PE. To further challenge our hypothesis, we repeated the KWALP20 partitioning experiments in *N*-methyl-PE and in *N*,*N*-dimethyl-PE phospholipids; these are, from a structural standpoint, intermediates between PE and PC (PC is *N*,*N*,*N*-trimethyl PE) (Fig. 4d, Supplementary Fig. S5c). Increasing methylation increased KWALP signal in the bilayer (Fig. 4d, e, Supplementary Fig. S5c, d), an effect similar to increasing PC/PE ratio, in agreement with our prediction.

In conclusion, our data indicate that HDs prefer mixing with TG instead of being in contact with membrane phospholipids. Because phospholipid packing is less compact in a monolayer compared with a bilayer^9^, and because a monolayer thickness is half the thickness of a bilayer, partitioning toward monolayers is favored as they expose HD proteins to TG (Fig. 4c). Increasing the monolayer phospholipid packing, for example by increasing the PC/PE ratio, increases HD-phospholipid interaction at the expense of HD-TG interaction. In this case, the peptide less efficiently discriminates the monolayer of the bilayer from the monolayer covering TG and therefore its monolayer accumulation is dampened.

### TG is responsible for the accumulation of HDs in monolayers

Our model postulates that KWALP accumulates to LD monolayers because it mixes with TG more favorably than with membrane phospholipids (Fig. 4a, b). To challenge this model, we altered the relative affinity of the peptide for phospholipids over the oil phase by changing the chemical nature of the oil. We chose an oil phase very different from TG, namely silicone oil, in an attempt to trigger major changes in oil-protein affinity. Silicone oil is chemically very different from TG but they both have a high surface tension with water^9^.

**Fig. 5:**
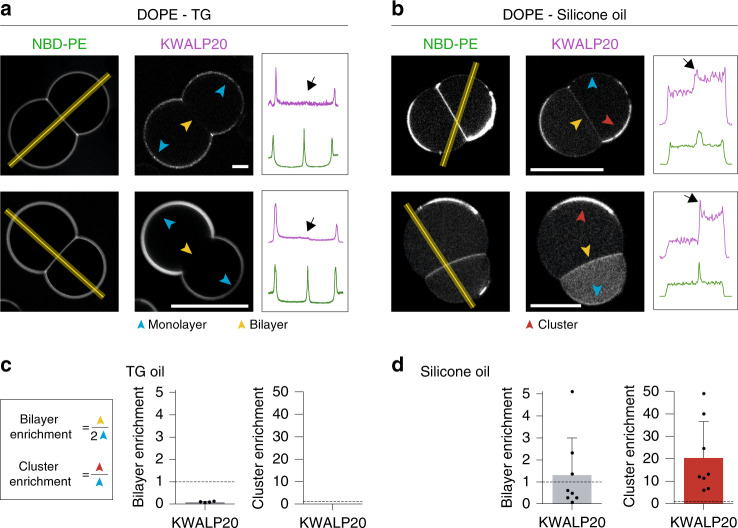
The distribution of KWALP HD depends on the oil chemistry. **a**, **b** Distribution of KWALP20 in DOPE DIBs. KWALP20 is labeled with Rh-B. Oil phase is TG (**a**) or silicone oil (**b**). Blue arrows indicate the monolayers, yellow ones indicate the bilayer, and red ones indicate cluster areas. Plot profiles are determined using the yellow lines. The bilayer signal is indicated by a black arrow. Scale bar: 20 µm. **c**, **d** Partition coefficient is reported in gray in TG (**c**) and silicone oil (**d**), as mean ± sd (*n* = 2 and 8 independent measurements respectively). A cluster enrichment coefficient (red) is determined for the experiment in silicone oil, and is shown as mean ± SD (*n* = 8 independent measurements). Each data point is shown as a black dot. Source data are provided as a Source Data file.

In PE DIBs made in TG, KWALP20 was absent from the bilayer, as described above (Fig. 5a, c). In contrast, by replacing TG with silicone oil, we systematically observed that KWALP20 was in the bilayer (Fig. 5b, d). Moreover, phospholipid clusters regularly appeared at the monolayer interface and they were enriched in the peptide (Fig. 5b, d). Outside these areas, the peptide signal was weaker at the oil-water monolayer interface. Our interpretation is that the peptide has a higher affinity for phospholipids than for silicone oil, and therefore it preferentially distributes to phospholipid-rich regions, i.e., the bilayer and the phospholipid clusters.

To further validate our findings, we repeated the same experiment with VAMP2, one of the SNARE components that binds to membranes with a transmembrane domain. In TG, VAMP2 was completely absent from the bilayer (Supplementary Fig. S5e, g) while in silicone oil it was in the bilayer and clustered with phospholipids at the oil-water interface (Supplementary Fig. S5f, h), exactly like KWALP20.

Altogether, our results suggest that HD-containing proteins partition to regions where they find the highest molecular affinity. They have more affinity for TG than for phospholipids, and therefore get enriched in sites offering easier access to TG.

### KWALP egresses membranes to accumulate in model LDs

The DIB system revealed the existence of an energy gap favoring the higher enrichment of HDs to monolayers, due to their preferential mixing with TG over bilayer phospholipids. Thus, when a nascent LD is formed in a bilayer, as during the early step of LD biogenesis (Fig. 1a), HDs would all preferentially relocalize to the nascent LD. Such behavior has been reported for many HD-containing proteins, including HPos, LiveDrop, or Oleosins^27,37,39,40,45^. We tested this hypothesis.

**Fig. 6:**
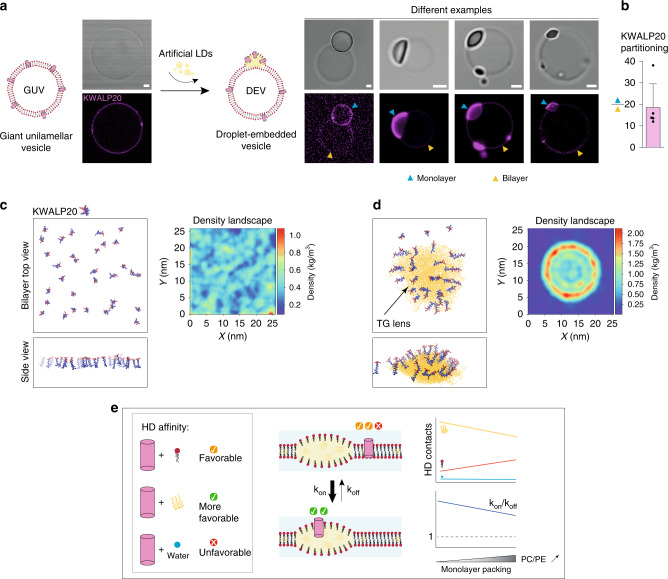
Nascent LDs are attractive to monotopic proteins. **a** Formation of DEVs-containing KWALP20 labeled with Rh-B: (left) KWALP20 is inserted in GUVs, during GUV electroformation or by mixing GUVs with the peptide; an example of the resulting KWALP20-interted GUV is shown. (right) KWALP20-containing GUVs are mixed with a TG-in-buffer droplet to generate droplet-embedded vesicles; several examples of the resulting DEVs are displayed: The peptide is labeled with Rh-B. The KWALP signals on the monolayer and the bilayer are respectively depicted by blue and yellow arrows. Strong accumulation happens at the monolayer. Image brightness is enhanced to improve monolayer viewing. Scale bar: 2 µm. **b** The ratio between monolayer and bilayer signals is plotted as mean ± SD (*n* = 5 independent measurements). Individual points are indicated. Source data are provided as a Source Data file. **c**, **d** Left: snapshot of molecular dynamics simulations of a bilayer with 32 KWALP20 peptides in parallel orientation, in the absence (**c**) and the presence (**d**) of a TG lens. Hydrophobic amino-acids are represented in blue and charged ones (lysines) in red. On the right of each simulation is displayed the average protein density profiles in the bilayer plane, averaged over the entire MD simulation (20 μs), in the absence (**d**) and in the presence (**e**) of the TG lens. **e** Basic model of how the different interactions of an HD favor its LD monolayer accumulation. HD interacts with TG, phospholipids or water; the monolayer packing regulates the contact between these species and tunes the reaction constant *k*_on_/*k*_off_.

To mimic the situation of a forming LD, we used the droplet-embedded vesicle system (DEV), which is a giant unilamellar vesicle (GUV) with TG droplets incorporated in between the bilayer leaflets^8^ (Fig. 6a). We incorporated KWALP into PC/PE (7/3) GUVs, during GUV electroformation or after, by mixing the GUVs with the peptide, (Fig. 6a, Supplementary Fig. S6a). Next, the KWALP-containing GUVs were mixed with TG-in-water droplets in order to generate DEVs (Fig. 6a). We found that the peptide was massively enriched onto the monolayer side, consistent with our predictions and with results obtained in DIBs (Fig. 6a, b, Supplementary Fig. S6b, c). Next, we used molecular dynamics simulations to account for possible size and curvature effects which are not recapitulated in DEVs or DIBs. We generated bilayers in which 16 or 32 copies of the KWALP20 peptides were all incorporated from one side of a DOPC bilayer. In the absence of TG, the peptides were randomly distributed (Fig. 6c). When TG was incorporated into the bilayer, first it spontaneously nucleated a lens, then almost all the peptides moved to the surface of the lens, as quantified by the peptide distribution profile (Fig. 6d). The peptides remained mobile and were able to transiently move to the bilayer region, indicating that the equilibrium is dynamic and no major kinetic barrier traps the peptides in the monolayer. The same result was obtained for KWALP28 peptides (Supplementary Fig. S6d). These results are consistent with the previous ones in the DEV and DIB systems.

Overall, these two sets of data indicate that the free energy of the system is lower when HD proteins are at the monolayer. These results reinforce the idea that HD-containing proteins can sense TG and accumulate at TG hotspots.

## Discussion

Protein-lipid interactions has a key role in membrane biology by controlling protein localization and functionality^14,28,31^. In particular, a variety of protein-phospholipid interactions are responsible for the localization of many proteins to specific organelles or membranes regions^28^. TG is not a membrane lipid but a bulk lipid. Our findings support that most HD proteins have a higher affinity for TG over phospholipids in a membrane environment (Fig. 6e). Consequently, under LD biogenesis conditions, HD-containing proteins would more favorably be recruited to sites of TG accumulation, instead of remaining in the ER bilayer (Fig. 6e). Helical hairpins, hydrophobic helices, and transmembrane domains not fully crossing the ER bilayer, would accumulate to nascent LDs. Even AH-containing proteins would do so, but to a lesser extent (Fig. 2f). In short, emerging nascent LDs in the ER would be hotspots that attract nearby HD-containing proteins. Controlling these stages of LD formation will be critical for defining the proteome of the emerging LDs^8,45,46,47^ and for keeping ER homeostasis.

While our results predict that HD proteins preferentially accumulate to LD monolayers, clearly not all ER HD-containing proteins target to LDs. Hence, there must be counteracting mechanisms that reduce and prevent unspecific HD-protein targeting to emerging LDs^5,6^. Amino acid composition of an HD might determine the HD-TG affinity and hence the ER-to-LD partitioning extent. For instance, the presence of charged residues in a HD may hamper HD-TG interaction, since embedding charges in a low dielectric milieu such as of a TG phase is unfavorable, and generally requires conformational changes to the protein or interaction with a protein of opposite charge. More generally, as we recently proposed for AHs^9^, there could exist sequence motifs tailored with an optimal affinity with TG.

The presence of HDs can perturb the structure of lipid bilayers, generating stresses that tend to reduce protein-phospholipid interactions, for example by clustering proteins, as predicted by theoretical studies and confirmed by molecular simulations^17,28,31^. LD monolayers have more phospholipid packing defects than bilayers^9^, and allow exposing TG to water molecules. During LD formation, the relocalization of HDs from the bilayer to a forming LD would reduce the stresses caused by the protein to the bilayer and possibly mask phospholipid packing defects at the LD monolayer. Such partitioning would be highly favorable as it would minimize energy on both bilayer and monolayer interfaces. Actually, even prior to LD assembly, transient TG clusters appearing in the ER bilayer^2^ may attract HD-proteins or, inversely, HD-proteins can trigger the clustering of TG molecules around them^48^, thereupon promoting LD nucleation and alleviating ER stress.

Finally, LD formation is stimulated by diverse physiological conditions such as excess nutrients or ER stress^11^. During ER stress, the formation of LDs may be stimulated in order to sequester damaged HD-containing proteins to be degraded, by macrolipophagy for example^49^. Indeed, in this case, proteins tend to expose hydrophobic sequences that would be favorably adsorbed to LDs. In this context, LD formation would serve as a protein quality control pathway maintaining ER proteostasis, a function different from the primary role of LDs in metabolism^50^. Accordingly, another mechanism triggering ER bilayer stress is the alteration of ER phospholipid composition^51^, especially when PC/PE ratio is decreased^10,52,53^. Here, our data bring important insights on how this ratio can modulate the partitioning of HD-containing proteins between ER and LDs: decreased PC/PE favors HD targeting and retention to the LD monolayer. Thus, by tuning PC/PE ratio, cells may be able to shift more HD-containing proteins from the ER bilayer to LDs, or vice versa, for degradation for instance. Along this analysis, increased PE/PC levels in liver is caused by dysfunctions of the phosphatidylethanolamine N-methyltransferase and associated with steatohepatitis^54,55^, a condition linked to LD formation. Based on our data, such PE/PC-induced steatosis may be related to abnormal ER-to-LD protein trafficking. In contrast to mammalain cells, Drosophila cells present high PE/PC levels under normal conditions^56^; therefore, the ER-to-LD partitioning extent of HD-proteins in these cell lines may strongly differ from mammalian cells.

In conclusion, our data connect various fields involving protein-lipid interactions, from basic membrane biophysics to membrane biology, lipid metabolism, and cellular proteostasis. Our findings highlight the attractiveness of LD surface for HD-containing proteins. Accumulating neutral lipids would be rapidly detected by proteins bearing these domains. Such non-selective detection is clearly prevented by cells by means to be discovered.

## Methods

### Material

HEPES, Kacetate, MgCl_2_, sodium phosphate monobasic, sodium phosphate dibasic, choloroform, trifluoroethanol, Octyl-ß-D-glucopyranoside were bought from Sigma Aldrich. DOPC (1,2dioleyl-sn-glycero-3-phosphocholine), DOPE (1,2dioleyl-sn-glycero-3-phosphoethanolamine), *N*-methyl-PE, *N*,*N*-dimethyl-PE, liver PI, Rhodamine-DOPE and NBD-DOPE were purchased from Avanti Polar Lipids, Inc. CAV1-GFP plasmid was purchased from Sino Biological (catalog no. HG11440-ACG). The following plasmids were gifts: YFP-CG2254 and YFP-CG9186 from Dr. Mathias Beller; GFP-Plin 1, GFP-Plin 1N, GFP-Plin 1C, mcherry-Plin 2 and mcherry-Plin 3 from Dr. David Savage; EGFP-ACSL3 plasmid from Dr. Joachim Füllekrug^57^; GFP-HPos from Prof. Albert Pol^40^. Cells were obtained from American Type Culture Collection and no contamination for mycoplasma was detected.

### Peptides and proteins preparation

RhB-KWALP peptides, RhB-ArfGAP1, RhB-GMAP-210 and NBD-CAV1-AH were synthesized by peptide 2.0 Inc., NBD-PL108 was made by Proteogenix SAS, and RhB-NS5A was synthesized by Eric Diesis. All the peptides were chemically synthesized and purified by reverse phase high-performance liquid chromatography (HPLC). Their purity was higher than 95%, as determined by analytical HPLC and their mass was confirmed by mass spectrometry. The amino-acid sequences of the peptides are:

KWALP20: RhB-GKKKLALALALALALALWWA-Amide

KWALP28: RhB-GKKKLALALALALALALALALALALWWA-Amide

ArfGAP1-AH: RhB-FLNSAMSSLYSGWSSFTTRAKKFAK

GMAP-210-AH: RhB-MSSWLGGLGSGLGQSLGQVGGSLASLTGQISNFTKDML

CAV1-AH: NBD-LFEAVGKIFSNVRINLQKEI

PL108: NBD-PPEKIASELKDTISTRLRSARNSISVPIAS

NS5A: RhB-SGSWLRDVWDWVCTILTDFKNWLTSKLFPKL-Amide

Plin proteins, CG2254, CG9186, CAV1, HPos, NS5A and ACSL3 were obtained from purified lipid droplets be using the following protocol. LD purification from Huh7 cells expressing fluorescently tagged LD proteins: cells from five 15 cm dishes were harvested, washed once in ice-cold PBS, and lysed using a 30 G needle in 1 ml of homogenization buffer containing 20 mM Tris and complete^TM^ protease and phosphatase inhibitors, at pH 7.5; for LD isolation, 1 ml of cell lysates was mixed with 1 ml of 60% sucrose in Tris-EDTA buffer supplemented with protease inhibitors, successively overlaid with 20, 10, and 0% buffered sucrose in an 5 ml Ultra-Clear centrifuge tubes (Beckman). The tube was centrifuged for 16 h at 100,000 G and 4 °C, using an SW60 rotor in a Beckman L8-70 centrifuge. The upper 300 µl fraction was collected from as the LD fraction.

Fluorescently labeled Arf1 was generated using an Arf1-variant in which the single cysteine residue of Arf1 was replaced with serine, and the C-terminal lysine was replaced with cysteine, yielding Arf1- C159S-K181C. In short, human Arf1- C159S-K181C and yeast *N*-myristoyltransferase were coexpressed in Escherichia coli supplied with BSA-loaded myristate. Cell lysates were subjected to 35% ammonium sulfate, and the precipitate, enriched in myristoylated Arf1, was further purified by DEAE-ion exchange. Eluted fractions of interest were concentrated in spin-column filters with a 10-kD cutoff (Millipore), and fluorescently labeled using Cy3-maleimide (GE Healtcare) according to the manufacturer’s protocol. To remove excess dye, samples were purified by gel filtration using a Superdex 75 column (GE Healthcare).

Oleosin1 lipid droplets were obtained from Arabidopsis seeds, provided by Dr. Martine Miquel.

Vamp2, Tsnare (complex of syntaxin1a and SNAP25) and Synaptotagmin 1 57–421 C277A, E265C (Syt1) were produced and purified by the team of Frédéric Pincet. The proteins Vamp2, Tsnare and Syt1 (solubilized in Octyl-ß-d-glucopyranoside (OG) micelles) were labeled with a fluorescent probe Atto-565 maleimide (Atto-tec, GmbH), according to the manufacturer’s instructions. Free-dye was removed by gel-filtration, using a Sephadex G25 column (PD-minitrap G25, GE Healthcare). Labeled-proteins were then purified in DOPC/DOPE 1:1 proteoliposomes (P/L 1:1000): DOPE and DOPC were mixed in a glass tube, then the chloroform was removed under an argon flow and the glass tube was left under vacuum for at least one hour. The resulting lipid film was rehydrated with the Atto565-protein solution during 30 minutes. The sample was then diluted 3 times to decrease OG concentration below its cmc and a dialysis was performed overnight in a 10 kDa Slide-A-Lyzer dialysis cassette (Thermo Scientific) in order to remove OG and keep the protein into liposomes. Final buffer was the following: 25 mM HEPES pH 7.4, 120 mM KCl, 1 mM DTT (with 0.5 mM CaCl_2_ for Syt1).

### Droplet interface bilayer formation

Unless mentioned, in vitro experiments were performed in HKM buffer: 50 mM HEPES, 120 mM Kacetate, 1 mM MgCl_2_ at pH 7.4. KWALP peptides were dissolved in trifluorethanol at 200 μM, and then diluted in HKM to get a final concentration of 10 μM. PL108 was solubilized at 50 µM in HKM, CAV1-AH at 10 µM in HKM (with 0.1% DMSO), GMAP-210 at 2 µM (with 0.1% DMSO, 16 mM urea, 80 µM DTT), ArfGAP1 at 8 µM in HKM (with 0.1% DMSO). NS5A was diluted in HKM to obtain a final concentration of 1 µM and 10% of trifluoroethanol was added to ensure of its folding. The proteins in LD (Plin 1, Plin 1 C, ACSL3, CG2254, CG9186, Oleosin 1, CAV1, HPos) or proteoliposomes (Syt1 57–421, t-snare, VAMP2) were used directly. DTT was added at a final concentration of 2.5 mM in case of aggregation.

Phospholipids (eventually with 0.2% of labeled-PE) were evaporated under a stream of argon to remove the chloroform. The resulting lipid film was then re-solubilized to the desired concentration (0.2% w/w) in trioctanoate (or silicone oil), strongly vortexed and sonicated for 10 min to ensure a complete dissolution. To form DIBs, buffer-in-oil emulsions were made using 10 μl HKM (or peptide/protein solution) dispersed in 100 μl of trioctanoate. This emulsion was strongly vortexed in order to let the protein relocalize to the surface of the droplets. Then, the same volumes of peptide/protein emulsion and lipids in oil phase were put together and the resulting emulsion was placed on a hydrophobic coverslip (glass coverslip #0 from Menzel Glaser, Braunschweig, Germany, which was covered by PDMS). The sample was let to equilibrate for 10 min and was then observed by confocal fluorescence microscopy (LSM 800, Carl Zeiss, Oberkochen, Germany), with a ×10 or oil-immersed ×63 objective depending of the size of the droplets. When the emulsion is poured onto the observation glass, droplets which are closer to each other spontaneously adhere, because of the poor solubility of the phospholipids in the oil phase, and form a bilayer. The final lipid concentration in the oil phase is then 0.1% w/w and the interfacial lipid composition is determined by lipid composition in this oil phase.

To study the effect of PC on the localization of KWALP or PL108 peptide in a dynamic way, the two peptides were both used at the same concentration of 50 µM in DOPE DIBs. Then, 5 µl of DOPC 0.2% in trioctanoate (containing 10% CHCl_3_) was added to the sample.

### Giant unilamellar vesicles formation

Phospholipids (DOPC/DOPE (7:3 or 6:4)) in chloroform at 2.5 mM were spread on an indium tin oxide (ITO)-coated glass plate. After chloroform evaporation, the resulting lipid film was then placed under vacuum for 1 h. The chamber was sealed with another ITO-coated glass plate. GUVs were grown by electroformation in a sucrose solution (0.1 g ml^−1^, ≈280 mosmol kg^−1^) with the following settings: 100 Hz, 1.25 V, for 1.5 to 2 h. They were then collected carefully with a Pasteur pipette, placed in a Eppendorf® tube and stored at 4 °C.

### Droplets embedded vesicles formation

Droplets were made using an oil-in-water emulsion: 20 μl of trioctanoate were mixed with 100 μl of HKM buffer. The solution is then sonicated to form small droplets. 10 µl of 20 µM KWALP peptide solution was added to 40 µl of a GUV solution, which were then incubated with 20 µl of droplets for 5 min. We also added KWALP to dried phospholipids prior the electroformation and this also led to the incorporation of KWLAP to GUVs, which were subsequently used to make DEV (Fig. 6). With both approaches, the DEV/KWALP sample was then placed on a glass coverslip pretreated with 10% (w/w) BSA and washed three times with buffer, and it was then observed by confocal fluorescence microscopy (LSM 800, Carl Zeiss, Oberkochen, Germany), with an oil-immersed ×63 objective.

### Electrical measurement

Aqueous droplets in oil were blown at the tip of micropipettes containing Ag/AgCl electrodes (connected to an Axopatch 200B amplifier—Molecular Device) and filled with electrolyte buffer. Micropipettes are made from borosilicate capillary (Harvard Apparatus, 1.0 mm OD×0.50 mm, ID×150mm) pulled with a micropipette puller (Sutter Instrument) to obtain tip with inner diameter of 2 µm. Before any use, tip of the micropipettes was treated dipping in a dimethyldichlorosilane solution to avoid capillary wetting by the aqueous droplets. Micropipettes were manipulated through MP225 and MP285 micromanipulators (Sutter Instrument). After blowing droplets at each micropipettes tip, 5 min are waited to allow monolayer formation, then micropipettes are moved to put into contact the two droplets to allow formation of the bilayer. Once DIB is stable, the electrical measurement was performed. This consisted in repeatedly imposing a 20 mV voltage step for 300 ms between the two sides of the DIB and measuring the resulting current. At the same time as capacitance measurements, images of droplets were acquired using a IDS camera mounted on an Olympus IX71 inverted microscope with a 20x objective to measure bilayer area of the DIB.

### Thickness calculation

The capacitance value C was obtained from the fitting by an exponential of the transient capacitive current at the beginning of the voltage step to determine its time constant. The thickness of the bilayer was then calculated assuming that the bilayer can be assimilated to a dielectric material using the relation: $e = \frac{{\varepsilon _r.\varepsilon _0.S}}{C}$ where *ε*_0_ is the permittivity of vacuum, *ε*_*r*_ the dielectric constant of the material (*ε*_*r*_ = 2.8)^58^ and S the surface of the bilayer calculated from images treated on ImageJ.

### Molecular dynamics simulations

To study protein distribution in nascent LDs, we carried out molecular dynamics (MD) simulations at the coarse-grained level using the MARTINI force field^50,59,60^ (version 2.2). First, we generated a system containing 2016 DOPC lipids, 625 triolein (TO) molecules, and approximately 83,000 water particles; the approximate size was ca. 27 × 27 × 18 nm. TO molecules were initially dispersed homogeneously in the DOPC bilayer, and phase-separated spontaneously to form an oil lens in the bilayer. The system was simulated for 20 μs, and its shape and properties did not change during the last 10 μs. Then, protein-containing systems were generated from the equilibrated lens system, inserting 16 or 32 copies of different transmembrane peptides. Peptides were always inserted in the bilayer region of the system. We used 2 similar peptide sequences, KWALP20 and KWALP28, both in parallel and anti-parallel orientation (i.e., half of the peptides pointing up and half pointing down). In total, we built 8 different protein-containing lens systems. For each system, MD simulations were carried out for 20 μs, and the last 10 μs were used for analysis.

To study the distribution of phospholipids in LDs, we carried out simulations of large nascent LDs, containing 18144 phospholipids (DOPC and/or DOPE), 7500 TO molecules, and ca. 1.9 million water particles; the system size was approximately 78 × 78 × 40 nm. We carried out 3 simulations: one with 100% DOPC, one with DOPC:DOPE 80:20, and one with DOPC:DOPE 60:40. Each simulation was carried out for 30 μs, and the last 20 μs were used for analysis.

All coarse-grained MD simulations were carried out with GROMACS (v2016.4) software^61^, using the leap-frog integrator and a time step of 20 fs. Non-bonded interactions were calculated with the Verlet neighborlist algorithm, with a Verlet buffer tolerance of 10^−6^ kJ mol^−1^ ps^−1^ and a cutoff of 1.1 nm; electrostatic interactions were shifted to zero from 0 nm, long-range electrostatics were calculated with the reaction-field method (εR = 15, εRF = ∞); Lennard-Jones potential was shifted to zero at the cutoff. The stochastic velocity rescaling thermostat^62^ with a time constant of 1 ps was used to maintain the temperature of the membrane (lipids and proteins) and the solvent separately at 300 K. Pressure was controlled semi-isotropically using the Parrinello–Rahman barostat^63^ with a reference pressure of 1 bar, compressibility of 4×10^−4^ bar^−1^, and a time constant of 12 ps.

Analysis of protein density was carried out with in-house software^17^ after re-centering the trajectory, using the center of mass of the largest TO cluster as the center of the simulation box.

Analyses of TO content in the bilayer, DOPC:DOPE contact fraction and mixing, and phospholipid distribution between bilayer and monolayer region were carried out with in-house software, freely available on our web site (https://mmsb.cnrs.fr/en/team/mobi-en/softwares/).

To study the effect of DOPE lipids on membrane thickness, we carried out all-atom simulations of pure DOPC and DOPC:DOPE 1:1 mixtures, using the CHARMM36 force field^64^ and the TIP3P water model^65^. Simulation boxes contained 100 lipids (50 per leaflet) and 5000 water molecules, and simulation time was 400 ns.

Simulations were carried out with the GROMACS 2020 software, using the leap-frog integrator and a time step of 2 fs. Non-bonded interactions were calculated with the Verlet neighborlist algorithm, with a Verlet buffer tolerance of 10^−6^ kJ mol^−1^ ps^−1^ and a cutoff of 1.2 nm. The PME algorithm^66,67^ was used for long-range electrostatics. The temperature was maintained at 298 K using the stochastic velocity rescaling thermostat^62^ with a time constant of 1 ps. Pressure was controlled with the semi-isotropic Parrinello–Rahman barostat^63^, with a reference pressure of 1 bar, compressibility of 4.5 × 10^−4^ bar^−1^, and a time constant of 10 ps. Analysis of mass density was carried out over the last 300 ns of the trajectories, with standard GROMACS tools.

### Circular dichroism

CD spectra were recorded over the wavelength range 185–250 nm, at 0.2 nm intervals and 20 nm min^−1^ scan speed, on a Jasco 815 spectropolarimeter (Jasco Inc., Easton, MD). Temperature was kept at 20 °C. Spectra measurements were performed in a 1 mm path length quartz cells from Hellma GmbH (internal volume 200 µl). Experiments were done either in TFE, 10 mM phosphate buffer pH 7.4, DOPC small unilamellar vesicles or TG emulsions with or without phospholipids (DOPC, DOPE, DOPC/DOPE 1:1). To prepare small unilamellar vesicles, DOPC in chloroform was put in a glass tube and the chloroform was removed using a stream of argon. Then the resulting lipid film was dried under vacuum for at least 30 min, and it was rehydrated with phosphate buffer and vortexed strongly. Finally, the lipid solution was sonicated to reduce the size of the vesicles. The oil-in-buffer emulsions were done by mixing 30 µl trioctanoate (eventually 0.2% w/w phospholipids) with 500 µl of buffer, and then sonicating the mixture. KWALP concentration was 20 µM and DOPC liposomes concentration was 1 mM. Data obtained were collected and processed using the software Spectra Manager®, and are then reported as molar ellipticity per residue (degree dmol^−1^ cm^2^ residue^−1^), given by:

$$[\theta ]_{{\mathrm{molar}}} = \frac{{100 \, \times \theta }}{{c \, \times l \, \times N}}$$

where *θ* is the recorded ellipticity in degrees, *c* is the peptide concentration in mol l^−1^, l is the cell path-length in cm and N is the number of residues of the peptide. In order to estimate the peptide secondary structure content, an analysis of CD spectra was done using CDPro software^68^.

### Statistics

Data analysis and representation were performed in Prism 7 (GraphPad Software, US). Information about sample size, errors bars and statistical tests are reported in each figure legend.

### Reporting summary

Further information on research design is available in the Nature Research Reporting Summary linked to this article.

## Supplementary information

Supplementary Information

Reporting Summary

## Data Availability

Data supporting the findings of this manuscript are available from the corresponding author upon reasonable request. A reporting summary for this Article is available as a Supplementary Information file. Source data are provided with this paper.

## Source data

Source Data
