# Supplementary material for: Triacylglycerols sequester monotopic membrane proteins to lipid droplets
Source: Nat Commun. 2020 Aug 7;11:3944. doi: 10.1038/s41467-020-17585-8 (PMC7414839; doi:10.1038/s41467-020-17585-8)
Supplement: Supplementary file 1 — Supplementary Information [file 41467_2020_17585_MOESM1_ESM.pdf]

Supplementary information

Triacylglycerols sequester monotopic membrane proteins to lipid droplets

Caillon et al.

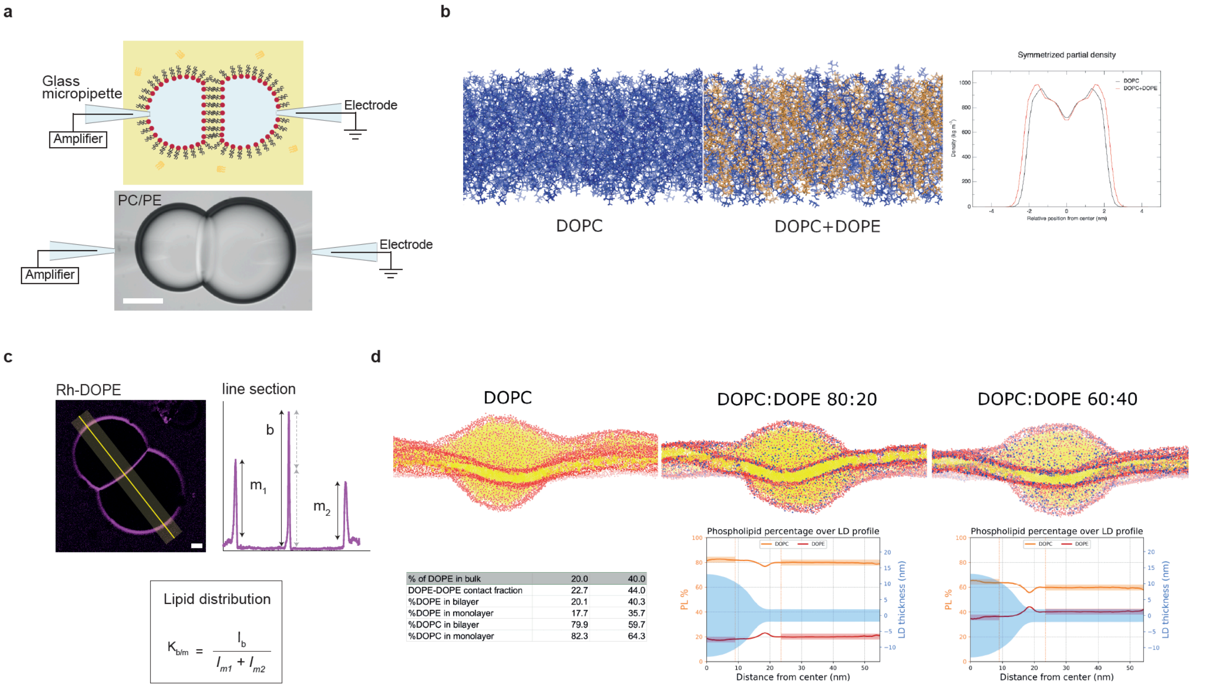

% of DOPE in bulk	20.0	40.0
DOPE-DOPE contact fraction	22.7	44.0
%DOPE in bilayer	20.1	40.3
%DOPE in monolayer	17.7	35.7
%DOPC in bilayer	79.9	59.7
%DOPC in monolayer	82.3	64.3

Figure S1. Droplet interface bilayers (DIBs) characterization, related to Figure 1.
(**a**) Illustration of the electrical measurements. Aqueous droplets were made at the tip of micropipettes containing electrodes, in oil containing phospholipids. Lipids adsorb at the oil/water interface and assemble as a monolayer. When two drops are brought together, they assemble to form a bilayer. The water phase is represented in light blue and the oil phase in yellow (neutral lipids, e.g. triglycerides). Electrical measurements are made between the two electrodes. DIB image microscopy is shown at the bottom. Scale bar: 100 µm. **(b)** In silico study of the impact of PE on bilayer thickness. Left, snapshots from all-atom simulation of DOPC and DOPC/DOPE (1/1) bilayers; DOPC is shown in blue, DOPE in orange. Right, density profiles for both bilayers against position of the phospholipids, showing an increase in overall bilayer thickness when DOPE is present. (**c**) Rh-DOPE distribution between the bilayer and monolayers of DOPE DIBs. The signal profile is determined using the yellow line and is shown on the right. The bilayer signal intensity is equal to the sum of the monolayers signal intensity for equal-partitioning. Lipid distribution is characterized by the partition coefficient $K_{b/m}$. Scale bar: 20µm. (**d**) Phospholipid distribution between a monolayer and a bilayer in contiguity, calculated from simulations of systems containing DOPC, DOPC/DOPE 80:20 and DOPC/DOPE 60:40 systems. The lateral size of the simulated boxes is ca. 80 nm for all three systems. DOPC head groups are represented in red, DOPE head groups in blue, TO in yellow. The average fraction of DOPC and DOPE in DOPC/DOPE systems is represented as a function of the distance from the center of the nascent LD, and reported in the table. In the diagram, the light blue shaded area shows the average shape of the nascent LD. DOPE-DOPE contact fraction indicates the probability that DOPE molecules contact with other DOPE molecules; if mixing is ideal, this corresponds exactly to the fraction of DOPE in the mixture (i.e., 20% or 40%).

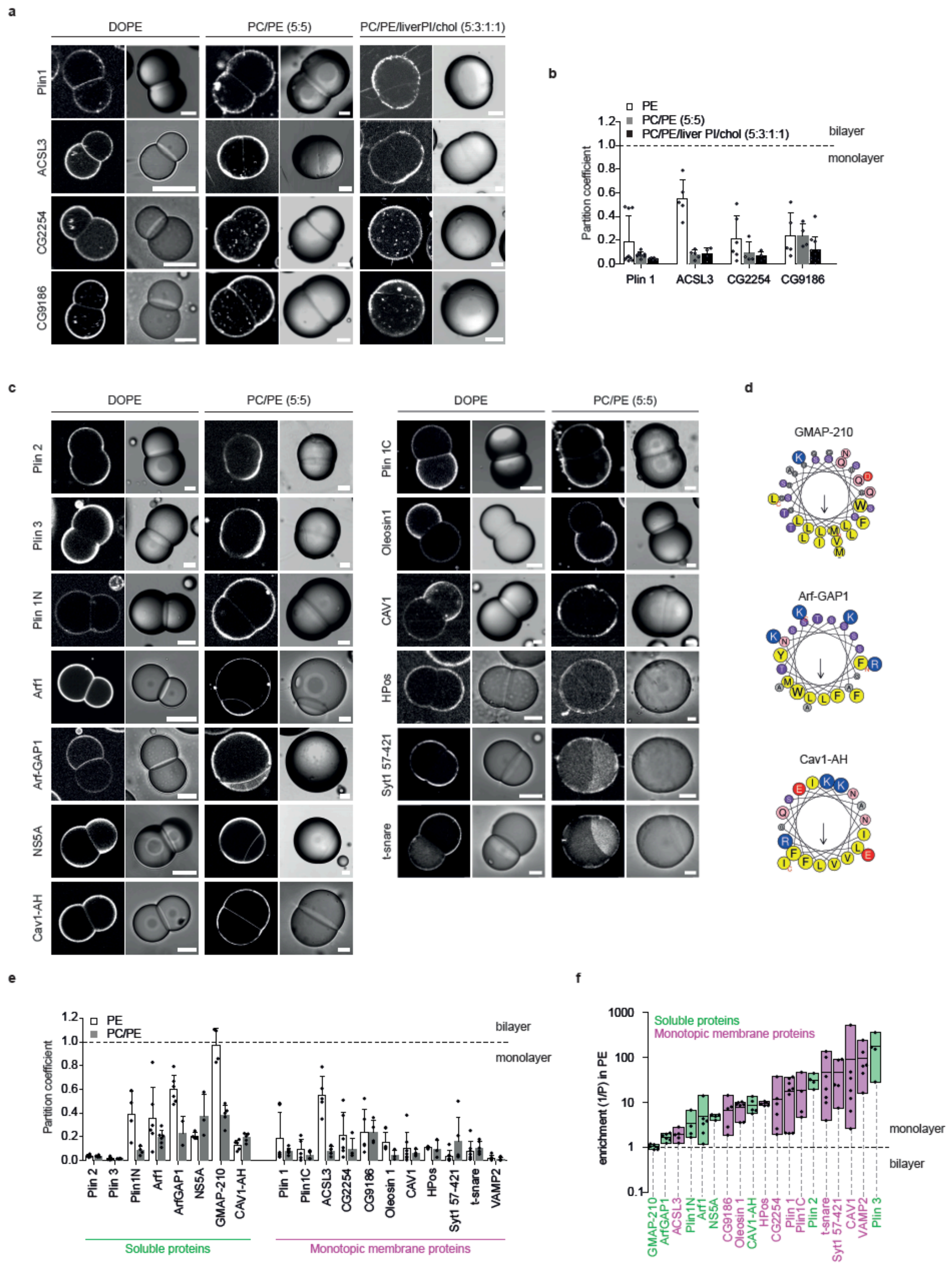

Figure S2. AH and HD proteins/peptides distribution in DIBs, related to Figure 2.
(**a**) Distribution of LD proteins (Plin1, ACSL3, CG2254, CG9186) in DIBs made of DOPE, or DOPC/DOPE (1:1), or DOPC/DOPE/liverPI/cholesterol (5:3:1:1). Scale bar: 20µm. (**b**) Partition coefficient of proteins from experiments shown in (a), shown as mean ± sd from at least 5 independent measurements. Individual points are shown as black dots. (**c**) Distribution of AHs- (left) and HDs- (right) containing proteins and peptides in DOPE and DOPC/DOPE (1:1) DIBs. Scale bar: 20 µm. HD-containing proteins were coming from purified LDs (Plin 1C, Oleosin 1, CAV1, HPos) or proteoliposomes (Syt1 57-421, t-snare). (**d**) Helical wheel representation of the peptides GMAP-210, ArfGAP1 and CAV1-AH, generated with HeliQuest[58]. (**e**) Partition coefficient of soluble (green) and monotopic membrane proteins (pink) in DOPE and DOPC/DOPE (1:1) DIBs, represented as mean ± sd. For each condition, n, independent measurements were done, 2 ≤ n ≤ 8. Individual data points are black dots. (**f**) Enrichment parameter in PE membranes for AH (green) and HD (pink) proteins shown as floating bars (bar limits, min to max values; central line, mean), 2 ≤ n independent measurements ≤ 8 for each protein. Individual points are shown as black dots. Source data are provided as a Source Data file.

a

Protein	Fragment	Location	Sequence	Nonpolar residues (%)	W, F (%)
Antigen-presenting glycoprotein - CD1D	302-322	PM, ER, endosome, lysosome	--MGLIALAVLACLLFLLIVGFT--------	86	9.5
Epidermal growth factor receptor - EGFR	646-668	PM, EC, N, ER, G, endosome	-IATGMVGALLLLLVVALGIGLFM-------	78	4.3
HLA class II histocompatibility antigen - DQB2	230-250	PM, ER, G, endosome, lysosome	-MLSGIGGFVLGLIFLGLGLII---------	67	9.5
Interferon gamma receptor 2 - IFNGR2	248-268	PM, ER, G	--VILISVGTFSLLSVLAGACFF--------	71	14.3
Fibroblast growth factor receptor 2 - FGFR2	378-398	PM, EC, G	--IAIYCIGVFLIACMVVTVILC--------	90	4.8
Furin - FURIN	716-738	PM, EC, G, endosome	-VVAGLSCAFIVLVFVTVFLVLQL-------	83	13.0
Low-density lipoprotein receptor - LDLR	789-810	PM, G, endosome, lysosome	--ALSIVLPIVLLVFLCLGVFLLW-------	91	13.6
Tumor necrosis factor receptor - TNFRSF1A	212-232	PM, EC, G	-VLLPLVIFFGLCLLSLLFIGL---------	86	14.3
Integrin beta 4 - ITGB4	711-733	PM	---FWWLIPLLLLLLPLLALLLLLCW-----	100	17.4
T-cell surface glycoprotein - CD3D	106-126	PM	---GIIVTDVIATLLLALGVFCFA-------	76	9.5
Epithelial cell adhesion molecule - EPCAM	266-288	PM	--AGVIAVIVVVVIAVVAGIVVLVI------	91	0
Insulin receptor - INSR	957-979	PM	--IIIGPLIFVFLFSVVIGSIYLFL------	83	17.4
Tyrosine-protein kinase receptor - TYRO3	430-450	PM	----VPVVLGVLTALVTAAALALIL------	86	0
Caveolin 1 - CAV1	105-125	PM, G	--------ALFGIPMALIWGIYFAILSFL--	86	19.0
Glycerol-3-phosphate acyltransferase 4 - GPAT4	156-176	ER	-----ISLRLTVLWGLGVLIRYCFLL-----	71	9.5
Glycerol-3-phosphate acyltransferase 4 - GPAT4	180-200	ER	-----IALAFTGISLLVVGTTVVGYL---	67	4.8
Diacylglycerol O-acyltransferase 2 - DGAT2	70-88	ER	--------VISVLQWVLSFLVLGVACS----	74	10.5
Diacylglycerol O-acyltransferase 2 - DGAT2	93-112	ER	-----YIFCTDCWLIAVLYFTWLVF------	85	25.0
Ancient ubiquitous protein 1 - AUP1	21-41	ER	---DCFLLLVLLLYAPVGFCLLVL-------	90	9.5
1-acylglycerol-3-phosphate O-acyltransferase 3 - AGPAT3	125-145	ER, N	-----ELLYVPLIGWTWYFLEIVFCK-----	76	19.0
1-acylglycerol-3-phosphate O-acyltransferase 3 - AGPAT3	317-339	ER, N	----ILLSPLFSFVLGVFASGSPLLIL----	74	13.0
Long-chain-fatty-acid-CoA ligase 3 - ACSL3	21-41	ER, Peroxisome, Mitochondrion	-----ILLYFIHFLISLYTILTYIPF-----	81	14.3
Lipid droplet-associated hydrolase - CG9186	166-186	ER	--------PLYSVFGYIFFSFFNFLPVWL--	81	33.3
Lipid droplet-associated hydrolase - CG9186	188-208	ER	--------LMLIQIYFLIFSIPRQFLGTA--	71	14.3
FI02989p - CG2254	19-40	ER	---------IYNIVLLVVDIVMLIVKFWLAI	86	9.1
Short-chain dehydrogenase/reductase 3 - DHRS3	9-29	ER, PM	--------LVMFPLQMIYLVVKAAVGLVL--	86	4.8
Short-chain dehydrogenase/reductase 3 - DHRS3	170-190	ER, PM	--------IVCLNSVLALSAIPGAIDYCT--	71	0
Short-chain dehydrogenase/reductase 3 - DHRS3	195-215	ER, PM	--------AFAFMESLTLGLLDCPGVSAT--	62	9.5
Short-chain dehydrogenase/reductase 3 - DHRS3	253-273	ER, PM	--------AVQLNQALLLLPWTMHALVIL--	76	4.8
Retinol dehydrogenase 10 - RDH10	3-23	ER	--------IVVEFFVVTFKVLWAFVLAAA--	86	23.8
**KWALP20**	-	-	---GKKKLALALALALALALWWA--------	**80**	**10**
**KWALP28**	-	-	---GKKKLALALALALALALALALALALWWA	**86**	**7.1**

PM: Plasma Membrane, N: Nucleus, EC: Extracellular, G: Golgi, ER: Endoplasmic Reticulum

b

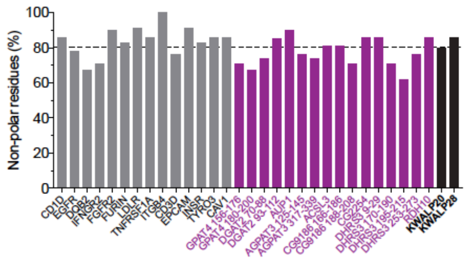

c

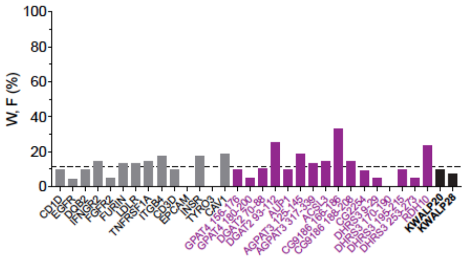

Figure S3. Comparison of KWALP peptide with the transmembrane domains of several proteins, related to Figure 3.

(**a**) Sequence alignment of KWALP peptide with the transmembrane domain of proteins found in bilayer-encircled organelles (black) and LD (purple). Hydrophobic amino acids are indicated in green, polar ones in black, and the positively and negatively charged in blue and red respectively. The transmembrane fragments, protein location and percentage of non-polar residues are indicated. The proteins CD1D, EGFR, DQB2, IFNGR2, FGFR2, FURIN, LDLR, TNFRSF1A, ITGB4, CD3D, EPCAM, INSR, TYRO3 were used in the analysis which allowed the design of transmembrane model peptides[72,73]; (**b, c**) Plot of the percentage of non-polar residues (b) or very hydrophobic residues W and F (c) for each fragment. The dashed line represents the mean of all these fragments.

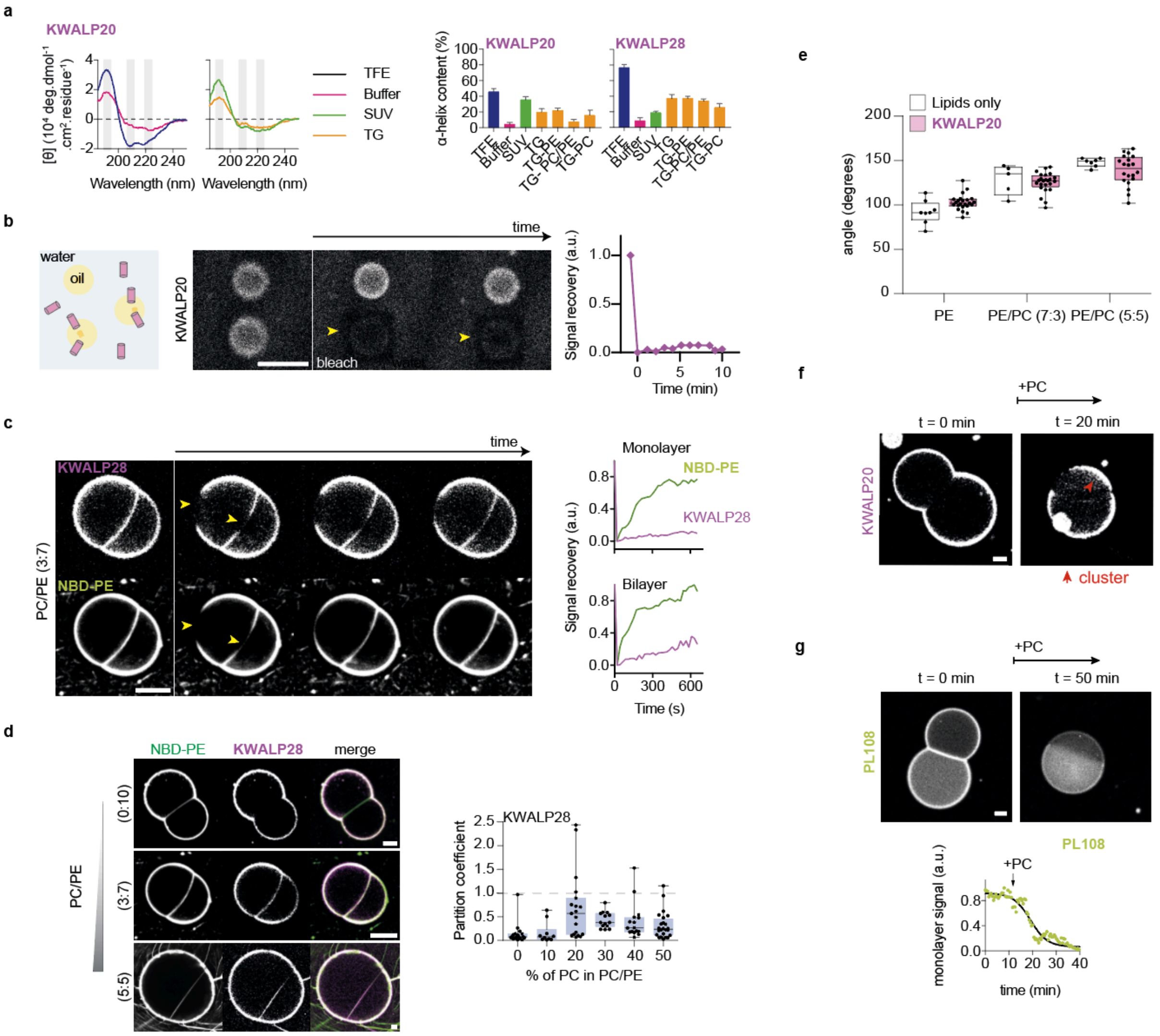

Figure S4. PC/PE modulates AH and HD localization, related to Figure 3.
(**a**) Circular dichroism spectra (top) of KWALP20 in various environments. Trifluoroethanol (TFE) is known to favor the α-helix structuration, the resulting graph (black) shows indeed the characteristics of an α-helix signal: a maximum around 192 nm, and two minima around 208 and 222 nm. These features are found also in buffer (purple), liposomes (green), TG droplets (blue), with a signal less intense. The spectra were deconvoluted to extract α-helix content (bottom). The result is the mean ± sd from 21 deconvolution programs. (**b**) Rh-KWALP20 in solution relocalizes to oil/water interface of TG-in-water droplets. The fluorescence of the bottom droplet is bleached (yellow arrow). After 10 minutes, no fluorescence recovery was observed from the bulk. Scale bar: 20 µm. (**c**) FRAP experiment showing the recovery of KWALP28 and lipid signals. Yellow arrows indicate the bleached areas. The signal recovery in the monolayer and in the bilayer is plotted against time (right). Scale bar: 20 µm. (**d**) KWALP28 in DIBs of varied DOPC/DOPE ratios. KWALP28 is labelled with Rh-B and phospholipids were reported by NBD-PE (0.2% of total phospholipids). Scale bar: 20 µm. The partition coefficient for each lipid composition is plotted (right) as box-plots. Sample size was n=19 for 0% PC, n=10 for 10% PC, n=19 for 20% PC, n=14 for 30% PC, n=16 for 40% PC and n=24 for 50% PC. (**e**) DIBs contact angle is represented as a function of DOPC concentration with box-plots. Sample size is n=8, 5, 7 respectively for PE, PE/PC (7:3) and PE/PC (5:5), in the absence of KWALP20, and n=23, 24, 20 in the presence of KWALP20. (**f, g**) Relocalization of KWALP20 (f) and PL108 (g) after addition of DOPC to DOPE DIBs. After 20 minutes, KWALP20 shows some aggregates (red arrows). Scale bar: 20 µm. The monolayer signal of PL108 is plotted against time (g). Box-plots are defined as follow: box limits, upper and lower quartiles; middle line, median; whiskers, minimum and maximum value. Individual points are plotted. Source data are provided as a Source Data file.

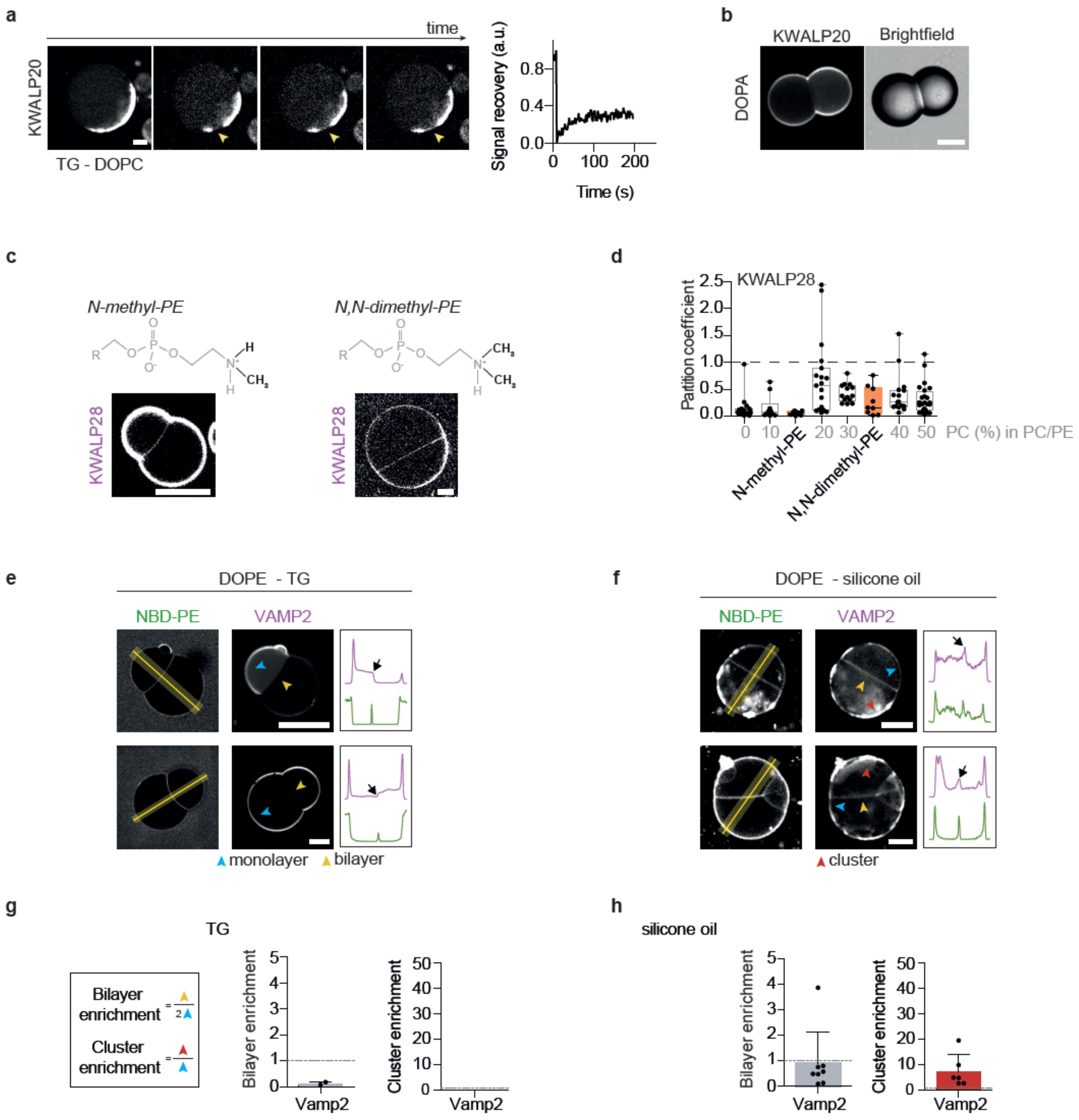

Figure S5. Influence of the phospholipid and oil composition on KWALP distribution, related to Figure 4, Figure 5.
(**a**) FRAP experiment on a cluster of KWALP20 in a DOPC droplet. Yellow arrow indicates the bleached area. Signal intensity was plotted against time. Scale bar: 20 µm. (**b**) KWALP20 in DOPA DIBs. Scale bar: 20 µm. (**c**) KWALP28 distribution in N-methyl-PE and N,N-dimethyl-PE DIBs. The headgroup methylation differs in these phospholipids. KWALP28 is labelled with Rh-B. Scale bar: 20 µm. (**d**) The partition coefficient of KWALP28 in DIBs of different compositions is represented as box-plots (box limits, upper and lower quartiles; middle line, median; whiskers, minimum and maximum value). Sample size is n=8 and 9 for KWALP28 in N-methyl-PE and N,N-dimethyl-PE respectively. Previous results with varying PC/PE ratios (Supplementary Figure S4d) are reported in light grey. Each data point is shown. (**e, f**) Distribution of VAMP2 in DOPE DIBs. VAMP2 is labelled with Atto565. The oil phase was TG (e) or silicone oil (f). Blue arrows indicate monolayers, yellow ones the bilayers, and red ones the cluster areas. Plot profiles are determined using the yellow lines. The bilayer signal is indicated by the black arrow. Scale bar: 20 µm. (**g, h**) The partition coefficient is reported in grey in TG (g) and silicone oil (h) as mean ± sd (n=2 and 8 independent measurements respectively). The cluster enrichment coefficient (red) was also determined for the experiment in silicone oil and is shown as mean ± sd (n=8 independent measurements). Individual data points are presented. Source data are provided as a Source Data file.

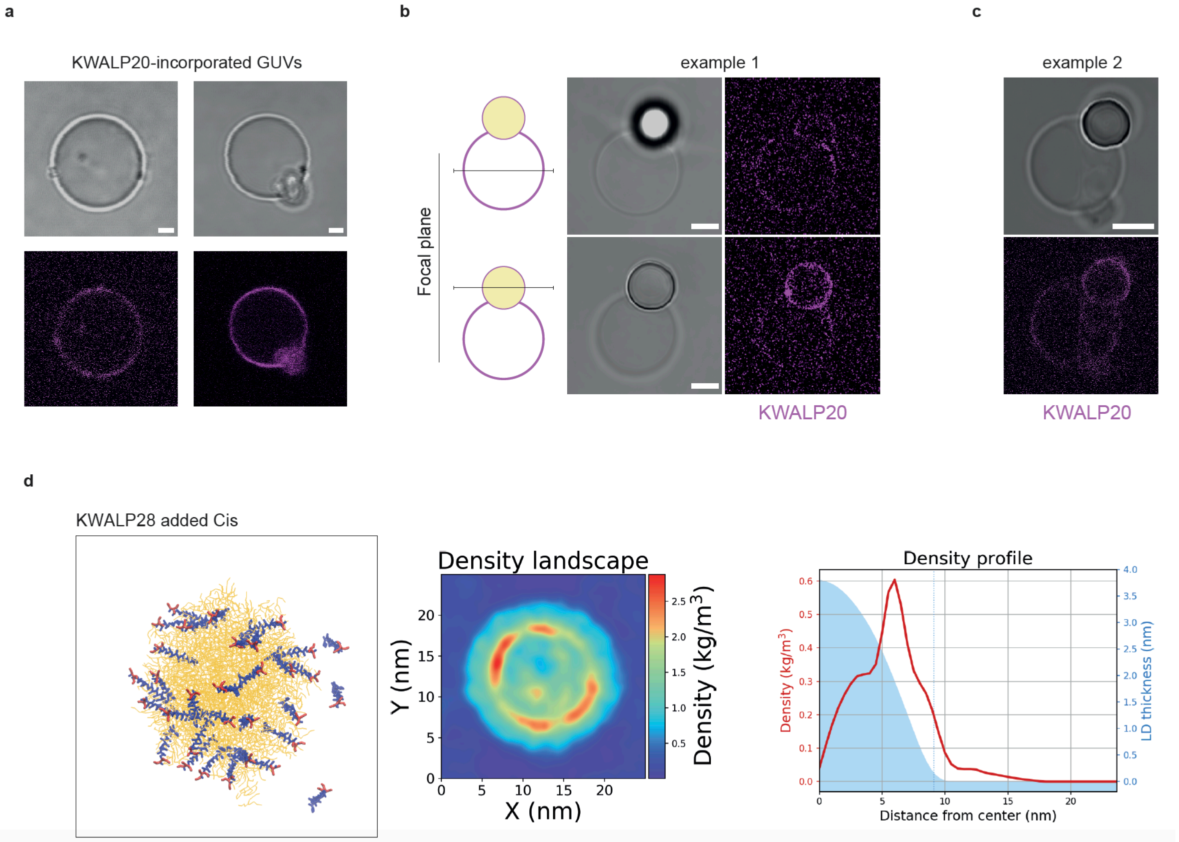

Figure S6. KWLAP favorably moves from the bilayer to nascent LDs, related to figure 6. KWALP20 peptide is labeled with Rh-20. (**a**) Example of KWALP20-incorporated GUVs made by electroformation. Scale bar: 2 μm **(b)** KWALP20 distribution in DEV system. Scale bar: 2 μm. The GUV and artificial LD are not in the same focal plane - images at their respective focal planes are displayed. **(c)** Another example of a KWALP20-DEV is shown. (**d**) Snapshot of a bilayer containing KWALP28 in parallel orientation in the presence of a TG lens. The protein density landscape in the membrane plane and the density profile as a function of the distance from the center of the TG lens are shown.
